# The Effect of Nanoparticles on the Structure and Enzymatic Activity of Human Carbonic Anhydrase I and II

**DOI:** 10.3390/molecules25194405

**Published:** 2020-09-25

**Authors:** Celia Cabaleiro-Lago, Martin Lundqvist

**Affiliations:** 1 Department of Environmental Science and Bioscience, Kristianstad University, 29188 Kristianstad, Sweden; Celia.Cabaleiro_Lago@hkr.se; 2 Department of Biochemistry and Structural Biology, Lund University, 22100 Lund, Sweden

**Keywords:** human carbonic anhydrase, nanoparticles, interaction, structure, kinetics

## Abstract

Human carbonic anhydrases (hCAs) belong to a well characterized group of metalloenzymes that catalyze the conversion of carbonic dioxide into bicarbonate. There are currently 15 known human isoforms of carbonic anhydrase with different functions and distribution in the body. This links to the relevance of hCA variants to several diseases such as glaucoma, epilepsy, mountain sickness, ulcers, osteoporosis, obesity and cancer. This review will focus on two of the human isoforms, hCA I and hCA II. Both are cytosolic enzymes with similar topology and 60% sequence homology but different catalytic efficiency and stability. Proteins in general adsorb on surfaces and this is also the case for hCA I and hCA II. The adsorption process can lead to alteration of the original function of the protein. However, if the function is preserved interesting biotechnological applications can be developed. This review will cover the knowledge about the interaction between hCAs and nanomaterials. We will highlight how the interaction may lead to conformational changes that render the enzyme inactive. Moreover, the importance of different factors on the final effect on hCAs, such as protein stability, protein hydrophobic or charged patches and chemistry of the nanoparticle surface will be discussed.

## 1. Introduction

Carbonic anhydrases (CAs) are found in species from all kingdoms and are categorized into eight distinct classes (α, β, γ, δ, ζ, η, θ, and ζ) [1,2]. The α-class is found primarily in vertebrates and is the only class of CA in mammals. The classes of CAs are an example of convergent evolution since they are distinct from each other in primary amino acid sequences and 3-D tertiary structures, but all catalyze the same essential chemical reaction [3]. Carbonic anhydrases are metalloenzymes that catalyze the important interconversion between CO_2_ and bicarbonate Equation (1). This reaction occurs non-enzymatically as well, but the reaction rate is too slow in a biological context.


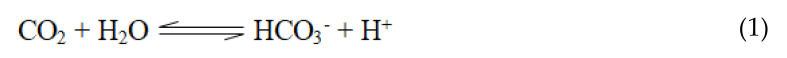


CAs in mammals are involved in many physiological and pathological processes such as respiration and CO_2_ transport, pH and CO_2_ homeostasis, biosynthetic reactions, calcification and tumor progression between others (more comprehensive information on physiological functions and pathogenicity can be found in diverse reviews) [4,5,6]. Due to this variety of functions, CAs are a potential target for therapeutic drugs in the treatment of diseases such as glaucoma, epilepsy, mountain sickness, ulcers, osteoporosis, obesity and cancer [4,5].

Nanoparticles (NPs) started to gain a lot of interest in the 1990s. NPs offer a much larger surface area per weight than larger sized particles. Moreover, some materials have different chemical properties when reduced to the nanosize compared to the bulk size of the material [7].

The combination of nanomaterial and biological macromolecules offers new possibilities for the design of functional materials. However, most proteins’ and enzymes’ functionality are dependent on their tertiary structure. Proteins that adsorb to a particle surface may undergo structural changes to optimize their interaction with the surface, which would lead to an unfunctional protein attached to the NP surface. The NP-protein construct has gained interest within the research community in the last two decades in three main areas: bio-nanotechnology, nanotechnology with medical applications, and nanosafety [8,9,10,11,12].

For CAs, two bio-nanotechnology applications have been mainly explored, the sequestration and capture of CO_2_ and the development of nano-inhibitors with high specificity and efficiency. Human CAs (and carbonic anhydrase from other organisms as well) have been combined with NP-based supports for the sequestration and transformation of CO_2_ into useful products as a measure to reduce the levels of CO_2_ in the atmosphere. Biocatalysts based on CAs are prepared via different approaches such as entrapment, covalent immobilization, or adsorption of the enzyme on different nanomaterials [13,14,15,16]. A few studies explore the possibility to develop specific nanoinhibitors for different variants of CA. Modification of the nanoparticle surface with known organic inhibitors leads to better efficiency and in some cases higher specificity towards a certain isoform of human CA [17,18,19].

This review will focus on two cytosolic CAs from the α-class, human carbonic anhydrase I and II (hCA I and hCA II) and their interaction with nanomaterials of diverse chemistry and size. hCA I and II are well characterized and different aspects for the proteins are thoroughly reviewed elsewhere [6,20,21,22,23]. However, the physiological function of hCA I is still unclear [4]. The focus here is the interaction/adsorption process and its consequences and therefore articles that concentrate on the final bio-nanoproduct or the covalent immobilization of the enzymes will not be covered. As Bhakta et al. wrote in their review from 2015, “One common problem observed in the literature is that while adsorption has been widely used to immobilize biorecognition elements to the surface of nanomaterials, only a fraction of those reports involve the corresponding adsorption study” [24]. In this review, we highlight what has been learned regarding the interaction between NPs and one of the hitherto most thoroughly characterized model proteins.

## 2. Survey

### 2.1. hCA I and hCA II

hCA I and hCA II are two of the 16 known mammalian CAs (15 human variants) [25]. Both variants are mainly expressed and found in red blood cells. They have homologous 3D-structures even though their sequence homology is just 60%. As with most of the CAs, hCA I and II coordinate a zinc ion in their active form. hCA II is one of the most efficient known enzymes with a k_cat_/K_M_ = 1.5 × 10^8^ M^−1^s^−1^ for the conversion of carbon dioxide to bicarbonate as shown in Equation (1) while hCA I has a lower efficiency with a k_cat_/K_M_ = 5 × 10^7^ M^−1^s^−1^ for the same reaction [26].

**Figure 1:**
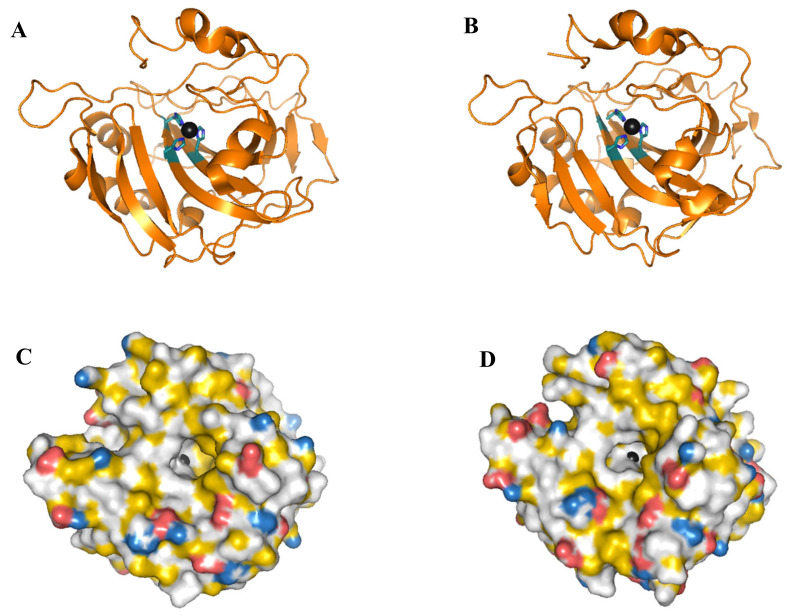
(**A**,**C**) hCA I, and (**B**,**D**) hCA II (wild type), shown as cartoon images (**A**,**B**) and space-filled images (**C**,**D**). The zinc ion is shown as a circle in black and the side chains of the three histidines that coordinate the zinc ion are shown in ball and stick. The lower figures show a spaced filled model of the proteins colored according to a YRB scheme [39]. (Red) oxygen atoms in D and E side chains. (Blue) nitrogen atoms in R and K side chains. (Yellow) part of the protein with high potential to form hydrophobic interactions. Images were prepared from pdb: 2cab [40] and 2ili [41] for hCA I and hCA II, respectively, using the PyMOL Molecular Graphics System, Schrödinger, LLC.

Figure 1 shows the outline of hCA I and hCA II tertiary structure. Their tertiary structures are similar with a β-sheet composed of 10 β-strands expanding throughout the structure. The active site is located in a deep cavity where a zinc ion is coordinated to three conserved histidines. One side of the active site shows a cluster of hydrophobic amino acids that facilitate the binding of CO_2_ [21,27].

hCA I and II have, in their native form, one cysteine each. The cysteines can be mutated to serines without affecting the enzymes catalytic capacity [28] and the pseudo wild types are frequently used for research purposes. From now on in this article, hCA II refers to the pseudo wild type unless otherwise stated. Despite their highly conserved tertiary structure, the 40% difference in the amino acid chain results in a difference when it comes to melting point and tolerance to urea and guanidine hydrochloride being hCA I more stable than hCA II [28,29,30,31,32,33]. Professor Bengt-Harald Jonsson’s research group has produced several different N-terminal truncated variants of the hCA II pseudo wild type. All the truncated variants they produced are less stable than hCA II [28,34].

hCA I and II (both wild type and pseudo wild type) unfold via a so-called molten globular state [34,35,36]. Molten globular state is a stable conformation between the native state and the unfolded state that retains most of the secondary structure of the native state. However, the tertiary structure is partially distorted and the molten globular state lacks enzymatic activity [34,37,38].

### 2.2. Common Techniques for the Study of hCA–NP Interaction in Solution

Diverse techniques have been used for the study of the interaction between NP and hCA I and hCA II in solution. Here, we describe shortly the different techniques focusing on the particular method or challenges in the study of the interactions of hCAs and NPs as well as which kind of information can be extracted from each technique (Table 1). Before we go into the different techniques, we want to highlight that it is important to think about what is measured with these methods. For some of the techniques (circular dichroism, fluorescence, etc.), care must be taken when interpreting the data since an observed change in measured signal can be originated from a partial change in all molecules or a change in a fraction of the sample while the remaining part stays unaltered.

Table 1 summarizes common techniques used to study particular aspects of the interaction between hCA I and hCA II with different nanoparticles (material and size for NP is specified in the table). Some techniques that have been marginally used are included in the table but not explained in detail in this section.

#### 2.2.1. Circular Dichroism Spectroscopy

Circular dichroism (CD) spectroscopy measures the difference between absorbance of left- and right-polarized light, respectively. The CD spectra is commonly divided into far-UV CD and near-UV CD. The far-UV-CD spectra can be used to investigate the extent of different secondary structure elements in a protein and it can also be used for following changes in secondary structure due to, for example, adsorption to an NP. Near-UV CD reports on the local environment around mainly the amino acid tryptophan. For a thorough review about CD, see Ranjbar and Gills [56].

**Figure 2:**
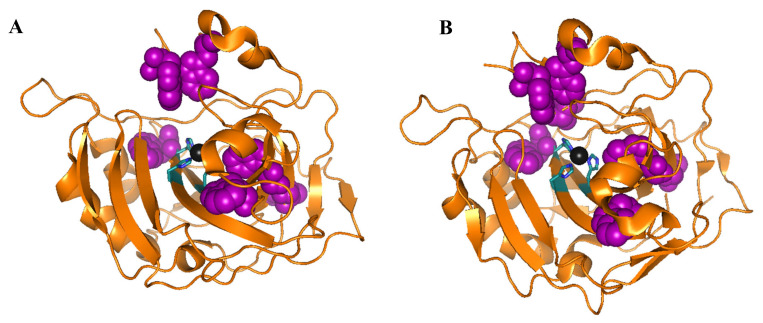
Ribbon structure with highlighted tryptophan side chains in purple for (**A**) hCA I (pdb:2cab) and (**B**) hCA II (wild type) (pdb: 2ili). Zinc ion is shown as a black sphere. Images were prepared using the PyMOL Molecular Graphics System, Schrödinger, LLC.

CD is one of the main methods to investigate the adsorption of hCA to NP. hCA I and II have six and seven tryptophans, respectively, which are spread in the native structure (see Figure 2). Prof. Jonsson’s and Carlsson’s research groups have characterized the individual contribution to the near-UV and far-UV CD spectrum of each of the seven tryptophans in hCA II which can be used to understand local changes in tertiary structure during protein denaturation/renaturation or adsorption onto surfaces. In near-UV CD measurements the tryptophans are the main contributors to hCA I and II near-UV CD spectra, i.e., measuring the near-UV CD spectra for hCA I and II will give information about local structural changes in the structures around the tryptophans. These residues also contribute to the spectra in the far-UV region, which makes the analysis of changes of secondary structure difficult for hCA I and hCA II [57].

#### 2.2.2. Nuclear Magnetic Resonance

hCA I and hCA II have been widely studied by nuclear magnetic resonance (NMR) and their individual backbone atoms have been assigned [58,59]. The main challenge to overcome in order to study protein adsorption on particles by NMR is that the formed protein–particle complex has a slow tumbling rate compared to the NMR time scale leading to peak broadening or complete loss of NMR spectral lines [60]. A combination of triple label variants and a water flip-back TROSY pulse sequence allows the study of the interaction between hCAs and particles in the low nanometer range [42,43].

#### 2.2.3. Fluorescence Spectroscopy

Many fluorophores can be used for investigating protein adsorption to NP due to their ability to report changes in their close environment [61]. When it comes to hCA, it is mainly three different fluorophores that have been used: 8-Anilinonaphthalene-1-sulfonic acid (ANS), 5-dimethylaminonaphtalene 1-sulfonamide (DNSA) and 5-((((2-iodoacetyl)amino)-ethyl)amino)naphthalene-1-sulfonic acid (1,5-IAEDANS). ANS has been used both to confirm structural changes in the protein after a fixed incubation time with nanoparticles [50], and to follow structural changes over time upon adsorption on NP [45,48,62]. DNSA is an extrinsic active site probe, hence, it can be used to report conformational changes at the active site of hCAs [50,63]. The 1,5-IAEDANS was used to map the binding place for the initial adsorption of hCA II to silica particles [52]. The fluorophore was covalently couple at different locations, one at the time, at the surface of hCA II. The fluorescence signal was then used to study the orientation of the enzyme at the particle surface.

#### 2.2.4. Enzymatic Assays

hCAs have, beside their biological activity, esterase activity that is commonly used for kinetic studies of the activity of these enzymes. The hydrolysis of esters, such as *p*-nitrophenyl acetate (NPA), by hCAs can be followed by absorption spectroscopy in the visible region due to the formation of a phenol (Equation (2)) [64,65]. The ability to hydrolyze esters is often used in in vitro assays to study the enzymatic activity of hCA I or hCA II due to their instrumental simplicity and slower reaction rates compared with the biological reaction:


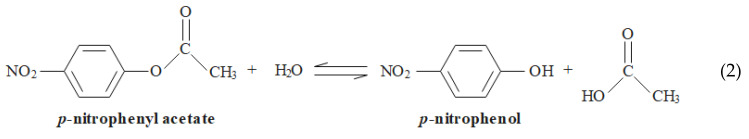


#### 2.2.5. Isothermal Titration Calorimetry

Isothermal titration calorimetry (ITC) is used to determine the enthalpy of binding in order to study enthalpic and entropic contributions in binding events such as protein adsorption on nanoparticles [66]. It also allows for determining the stoichiometry of the complexes formed. It has been frequently used in the study of metal, substrate and inhibitor binding to CAs [67] but ITC has also been used to study the interaction between CAs and nanoparticles [45,53,68].

#### 2.2.6. Mass Spectrometry

Mass spectrometry (MS) is a powerful method for identifying molecules in mixtures. MS can be used, for example, to identify (and quantify) proteins. MS has been extensively used during the last three decades to identify the proteins in the so-called “protein corona”, hence, the layer of specific proteins, from a complex biological solution, that adsorb to a specific nanoparticle [69]. MS can also be used to investigate how a protein binds to a nanoparticle by either using hydrogen/deuterium exchange [70] or limited proteolysis in combination with MS [51,71].

### 2.3. hCAs and NPs

We want to start this section with our interpretation of the concepts interaction and adsorption when it comes to proteins and nanoparticles. Processes in which the different objects spend a short time associated together and thereafter separate are defined as transient interaction. Adsorption occurs when the two objects stick together for a significant length of time. Adsorption is characterized by really slow desorption rates compared with adsorption rates. Proteins that adsorb to a surface often undergo structural rearrangements in order to optimize their binding to the surface. Initially, the protein can establish transient interactions between the protein surface and the particle surface. After the initial interaction, and depending on the residence time on the surface, the protein can start to unfold to establish new and stronger interactions with the surface leading to stable adsorption [72,73].

Most of the studies regarding the interaction between hCAs and NPs use silica and polystyrene nanoparticles of different sizes (Table 1). Within the pH range studied silica, carboxy-modified and plain polystyrene hold a negative surface charge while amino modified polystyrene nanoparticles are positive.

#### 2.3.1. hCAs and Interaction with/Adsorption to NPs

hCAs were among the first proteins to be characterized when it comes to their interaction with NPs. Billsten et al., Karlsson et al., and Lundqvist et al. all characterized the interactions with different methods and research focuses. Billsten et al. used ellipsometry to show that hCA II and two truncated variants of hCA II adsorb to silica NPs [50]. These early results were later confirmed, along with mutants of hCA II, with size exclusion chromatography, NMR and analytical ultracentrifugation [42,43,44,48,51]. hCA II, and especially hCA II mutants and the truncated variants of hCA II, absorb onto surface of silica NPs. When there is an excess of particle surface in the sample, all hCA IIs are adsorbed to the particles within 24 h [43,49,50].

hCA I, which is more thermodynamic stable than hCA II, shows a different behavior in its interaction with silica NPs. Lundqvist et al. showed that hCA I interacts with the silica NP surface, however, it does not adsorb (stay a long time at the particle surface) [43]. hCA I has instead a transient interaction. The protein goes on and off the particle surface and gradually achieves a longer residence time at the surface. However, the interaction with silica NP affects the native structure of hCA I and for small nanoparticles, 6–15 nm, the size of the particle matters for the effect. The presence of 15 nm silica particles has a significantly larger effect on the native structure of hCA I than the 6 nm particles [43].

Assarsson et al. used ITC to study the interaction between hCA II and polystyrene NP with different surface modification (carboxyl, amino, and unmodified) [53]. The unmodified and carboxyl-modified NPs have negative ζ-potential while the amino-modified NPs have positive ζ-potential. ITC measurements show clear heat signals when hCA II is titrated into plain and carboxyl-modified polystyrene NPs but not for the amino-modified polystyrene NPs, indicating that hCA II adsorb to the negatively charged NPs but not to the positively charged ones. The dynamic radius of the complex NP-hCA II, as seen by DLS, increases as the protein concentration increases for carboxyl-modified NP but not for amino-modified NP. Moreover, determination of the concentration of bound and free protein after incubation of hCA II with NP and subsequent filtration resulted in similar adsorption curves which confirm adsorption of hCA II on negatively charged NPs. Studies conducted at pH 8.2, at which the hCAs’ variants are negatively charged, show that the proteins (hCA I, hCA II and trhCA II) all adsorb to the surface of carboxyl-modified polystyrene NP. This is a clear indication that the hydrophobic effect has a strong contribution to the adsorption of the proteins on the carboxyl modified NP.

Furthermore, ITC results for the titration of hCA II (pI = 7.6) on carboxyl modified NP at pH 6.8, 7.4 and 8.2 show that the net charge does not affect the extent of adsorption, which supports that the hydrophobic effect plays an important role in the adsorption process. On the other hand, the charge of the protein affects the adsorption behavior of hCA I. Near the pI for hCA I (pI = 6.6), high protein coverage is obtained which decreases as the pH increases and the protein becomes more negative. Thus in the case of hCA I, electrostatic repulsions between proteins at the surface or with the particle counteract attractive interactions [46].

The size of an NP, of a specific material, may also affect how a protein interacts with it. This has been shown by Nasir et al. for hCA I and trhCA II interacting with carboxyl-modified polystyrene NP using ITC. trhCA II adsorbs on NPs with sizes 26, 49 and 94 nm while adsorption of hCA I could not be confirmed for the 49 and 94 nm particles. The ITC results suggest also a different packing of the proteins at the particle surface. The protein layer formed by trhCA II has a higher surface density than the protein layer formed by hCA I. The observed effects were explained considering the interprotein interactions at the NP surface. A high curvature reduced the interprotein interactions leading to a high packing if the protein does not lose its native form on the surface [45].

Other NP and CAs from other organisms have been investigated and CA adsorption on, for example, gold NPs with different surface stabilization was confirmed but this will not be covered in detail in this review [53,68].

#### 2.3.2. Characterization of the Binding Site(s)

The adsorption process of a protein to a surface is governed by a combination of different forces. The process can be divided into different steps: bulk diffusion of the protein towards the surface, docking at the surface, conformational changes at the surface and lateral diffusion/interactions at the surface [74]. Docking at the surface is driven by specific interactions between protein and surface, i.e., specific area(s) on a protein interact and bind to a specific surface. The identification of which part of hCA II and variants of hCA II initially binds to silica NPs has been thoroughly investigated by Prof. Carlsson’s and Prof. Jonsson’s research groups. The primary binding site on hCA II, when it adsorbs to silica NP, has been characterized. Karlsson and Carlsson used seven site-directly fluorophore-labelled variants of hCA II to determine the primary interaction area at different pH [52]. They showed that at pH under pI for hCA II (7.6) [46], it is the region around position 10 that is the dominating adsorption region. At pH under pI, this region is positively charged. However, when the pH is increased, the histidines in that area are deprotonated, the region loses its net positive charge, and an area around position 37 (which contains several lysines and arginines that have higher pK_a_ values than histidine) also starts to bind to the silica NP surface. The area around position 37, at pH ~8–9, can be described as slightly positive compared with other areas of the protein and is the base of the positively charged area of a dipole vector through the protein [52]. At pH, above 9 hCA II does not have a preferential binding site, however, it still adsorbs to the silica NP surface [52]. From this study, we can conclude that at pH values for which the histidines are protonated, the electrostatic forces will be the driving forces for hCA II adsorption to silica NPs. With increased pH, the importance of the electrostatic forces will decrease and hCA II will go from having a specific binding site to having an unspecific binding to the silica NPs.

The primary interaction site on hCA II when it adsorbs to silica NPs at pH ~ 8.5 was confirmed by Lundqvist et al. [51]. In the study, limited proteolysis was used followed by peptide detection with mass spectrometry to be able to identify which part of the protein was bound to the particle surface. Lundqvist at al. also showed that a mutant of hCA II, S56C, which is less stable than the wild type, initially binds to the silica NP in the same way as the wild type.

hCA I has been much less studied when it comes to characterizing the docking area to silica NP surface. The different behavior of hCA I compared to hCA II when it comes to its adsorption to silica NP surface suggests that hCA I probably has many unspecific interactions sites all around the structure and not any specific interaction point as in the case for hCA II.

#### 2.3.3. hCAs and Structural Changes upon Adsorption

Numerous methods have been applied to investigate the structural changes hCA undergoes after adsorption to NP, CD [43,45,48,49,50,51,53], NMR [42,43,44], limited proteolysis [51], and fluorescence spectroscopy [45,48,49,50]. The findings will be summarized and discussed here.

hCA I, as already discussed, does not readily adsorb to silica NPs, it rather interacts with the surface and then detaches out into the bulk again (see Section 2.3.1). NMR studies showed that with time, NMR signals decrease, indicating that hCA I residence time at the particle surface increases. When hCA I is bound to the nanoparticle surface, it will tumble along with the NP which will make it “invisible” in NMR due to the slow tumbling rate. However, even after 5 days together with silica NP, a native spectrum is observed for hCA I even though the signal intensity is greatly reduced [43]. That the extended residence time for hCA I at the silica NP surface results in structural changes is evident from CD studies [43], which show a gradual decrease of the native signal both for far- and near-UV CD with time. The changes in the native structure of hCA I caused by silica NPs are also obvious in NMR experiments in which spectra were recorded first with silica NPs present and then after the NPs had been removed from the system [44]. The recorded spectrum showed that structural changes in hCA I’s structure remained even after the particles had been removed from the sample. Even more striking is that hCA I was 100% active after the silica particles had been removed (which will be discussed later). All the results reviewed so far in this section are from experiments in which the NP surface area was in excess compared to the protein.

Nasir et al. studied the interaction between hCA I with silica particles, in conditions in which the protein was in excess over the NP surface area [45]. They followed the structural changes using ANS as a reporter molecule. The hydrophobic probe, ANS, for which the emission spectrum intensity increases with a blue shift of the maximum when it goes from a hydrophilic to a hydrophobic surrounding, is known to bind to molten globular forms of hCA [75,76]. With hCA I in excess over the NP surface area, only the largest investigated silica NP, 90 nm, showed any effect on the proteins structure with time. This is in line with Lundqvist et al.’s investigation [43], which showed that larger particles affected the native protein structure more at the same ratio between particle and protein surface area. The absence of increase in ANS fluorescence for the smaller silica may arise from three factors: the curvature of the NP, the low affinity of hCA I to the surface and the rapid exchange between bound and free molecules since the protein is in excess. Nasir et al. also studied hCA I interaction with different sized carboxylated polystyrene NP [45]. Near-UV CD experiments indicate that the conformational changes induced by NP are different to the ones induced by silica nanoparticles.

The evidence for hCA II adsorption on NP has already been discussed (see Section 2.3.1). The thermal stability of the enzyme has been shown to improve in the presence of functionalized silica particles. The effect was linked to structural changes upon adsorption and/or confinement of the unfolded enzyme on the particles that prevent hCA II thermal aggregation [54,55]. hCA II and different mutants and truncated forms of hCA II have been investigated regarding their structural changes upon adsorption to silica and polystyrene NP. The rate at which hCA II and different forms of hCA II undergoes structural changes is connected to the specific variants thermostability [48,50]. hCA II and destabilized variants of hCA II undergo large structural changes after adsorption to silica NP and the structural rearrangement at the particle surface is a process that goes on for days as evidenced by CD, limited proteolysis followed by MS, and NMR [42,43,48,49,50,51].

hCA II´s ability to adsorb to silica NP and undergo structural changes when bound to the surface is best demonstrated in Nasir et al.’s work [45]. The change in conformation of a truncated variant of hCA II in excess to the silica nanoparticles was followed by changes in ANS fluorescence intensity over time. Even in a situation in which the protein is in excess compared to the available surface, truncated hCA II adsorbs to the surface and starts to undergo structural changes. Unlike hCA I, for which only the sample with the largest silica NP gives increased fluorescence intensity, all three sizes of the silica NPs together with the truncated hCA II generate an increase in ANS fluorescence intensity over time. An NP size dependence can be observed where the largest particles (90 nm) give rise to a significant increase in fluorescence intensity. The ANS data reveal that upon adsorption, there is a “fast” first structural change which leads to a fast increase in ANS fluorescence intensity followed by a slower process that leads to more mild increases in fluorescence signal. The time frame for the structural rearrangement for truncated hCA II adsorbed to silica is within hours to days and the larger particles give rise to larger structural changes in the same time frame.

In the same article, the adsorption of truncated hCA II to different sized carboxylated polystyrene NP was also investigated. The protein shows a different behavior in the presence of polystyrene NP compared to silica NP. The structural change happens in a time frame of milliseconds to seconds at the NP surface. Moreover, the size dependence is opposite. The particles that give rise to extended adsorption of conformational changes are the smallest ones and the effect decreases as the size increases. The difference in time frame for the structural changes that the protein undergoes on the surface and in the NP size dependence between adsorption on silica compared to polystyrene NP surfaces was hypothesized to come from the different balance of forces that drive the adsorption of hydrophilic NPs compared to the adsorption of hydrophobic NPs [45].

#### 2.3.4. Activity

hCA I and II are efficient enzymes whose primary function is the conversion of carbon dioxide to bicarbonate. However, hCA I and II can also catalyze a range of different reactions [22]. In particular, the hydrolysis of esters [38,64,65] has been frequently used for the study of the effect of the interaction between hCAs and NPs on the activity of the enzymes. An activity assay will reveal if the interaction between a CA and a NP results in decrease, maintained or increased activity. Loss of activity can be caused by different means; e.g., for structural rearrangements in the protein, which affect the structure of the active site, blocking off the access to the active site by the NP or a combination of the two, i.e., care must be taken in interpreting activity data and preferably complementing structural methods should be used [19]. Inhibitory [17,19,77], activating [78] and multivalent [18] effects have been reported for surface modified nanoparticles. However, in general the observed effect is due to the specific molecule immobilized at the surface of the particle and not directly linked to that the nanosize of the carrier.

Using silica particles, Billsten et al. used an extrinsic active-site probe to estimate the accessibility to the active site for hCA II and truncated variants of hCA II in the presence of 9 nm silica particles [50]. hCA II showed a slight decrease in accessibility for the probe to the active site after overnight incubation with the particles while the two truncated variants had no accessibility. These data are supported by Karlsson et al. in studies where the esterase activity was used to investigate the effect silica NP had on the activity of hCA II and mutants of hCA II immediately after and at different time points after mixing with silica NP [48]. hCA II loses ~30% activity over a time frame of 24 h. The S56N mutant of hCA II, which has similar stability as the truncated variants and much lower than wild type, lost 80–90% immediately after mixing and 100% after ~15 h. This suggests that protein stability and loss of activity upon adsorption on NP is coupled. Similar inhibitory effects of bare silica nanoparticles have been reported by Touisni et al. [18]. Interestingly the effect of the nanoparticles on hCA II activity varies non-linearly with the amount of covalently attached inhibitors on the silica surface suggesting a multivalent effect of the ligands in the inhibition process [18].

hCA I has a more complex interaction with silica particles than hCA II and variants of hCA II as already mentioned. Bare silica causes negligible inhibition upon short incubation (15 min–2 h) that confirm the weak interaction between hCA I and silica particles [18]. Since hCA I does not really adsorb to the NP surface over a time frame of at least 7 days, it is in a constant exchange between free form in the bulk and associated form at the NP surface. Lundqvist et al. investigated how the presence of silica NPs affected hCA I esterase activity [44]. The measured activity decreases slowly with longer incubation times which is in line with the before-mentioned theory that hCA I gets longer surface-time with increased incubation time. The silica NPs affect both the hCA I structure and activity in the presence of silica NP. However, if the NPs are removed (together with the hCA I bound to their surface) two interesting observations can be noticed. First, the remaining protein in the supernatant is 100% active. Secondly, some of the structural changes in hCA I caused during its time at the silica NP surface remain after the particles had been removed according to NMR. Interestingly, some of the larger remaining structural changes, according to NMR, are located just under the active site but the protein is still 100% active. This indicates that the decrease in activity observed for hCA I in the presence of silica NP may be due to the blocking of the active site upon adsorption.

The study of the effect of polystyrene NPs by Assarsson et al. shows that hCA II does not adsorb to amino-modified polystyrene NP at pH around the protein isoelectric point [53]. However, it readily adorbs to plain and carboxyl-modified polystyrene particles (from ITC data) and its secondary structure is affected by the adsorption to the surface (from CD data). It is then not surprising that the presence of plain or carboxyl-modified polystyrene NP inhibits hCA II activity. At an excess of plain and carboxyl-modified polystyrene NPs, the activity goes down to 0 while samples that are treated with amino-modified polystyrene NPs remain 100% active. The inhibition of the activity most probably is caused by the structural rearrangement the protein undergoes when adsorbed to the surface which may disrupt the delicate structure of the active site. However, it cannot be ruled out that the inhibition of the activity comes from the blocking of the access to the active site when hCA II adsorbs to the surface. The exact binding site and structural conformational change has not yet been characterized for the hCA II:polystyrene NP case.

Assarsson et al. continued to study the interaction between hCA and polystyrene NPs [46]. hCA I, hCA II and trunc17 hCA II adsorption to and activity in the presence of different sized carboxyl-modified polystyrene NPs at different pH were investigated. Their study showed that pH and particle size had different effects on the activity for the three different proteins. The quenching of the activity for hCA II was not affected by pH (7.4–8.2 investigated) or particle size (25–114 nm investigated). However, pH is a factor on the effect of the particle size on the deactivation of tr17 hCA II and hCA I. tr17 hCA showed no dependence of the particle size at pH 7.4, however, at pH 8.2, the particle size mattered. Finally, the particle size matters for the activity for hCA I at both pHs investigated. The effect observed is correlated with the amount of protein adsorbed to the NP surface, i.e., hCA variants that readily adsorb on to NPs (hCA II) lose their enzymatic activity while hCA variants with little adsorption (hCA I) show a milder effect on the enzymatic activity [46].

Polymer modified-metallic nanoparticles have also been explored. Assarsson et al. has shown that hCA II activity is affected by poly(acrylic) acid (PAA) and poly(sodium styrene sulfonate) (PSS)-modified gold surfaces [53]. Gold surfaces modified with poly(allylamine hydrochloride) (PAH) on the other hand do not affect hCA II activity. This is in line with the findings for hCA II and polystyrene NP since PSS and PAA are negatively charged while PAH is positively charged. PSS is a strong polyelectrolyte which means that it is fully charged in solution while PAA is a weak electrolyte (hence just partially charged in solution). This is also in line with the observation with polystyrene NPs for which the more negatively charged carboxyl-modified NPs affect hCA II activity to a higher degree than a corresponding sample with plain polystyrene NPs (which are less negatively charged) for samples with similar particle surface areas [53].

## 3. Conclusions

Detailed characterization of how a specific protein interact with/or adsorb to a specific nanoparticle is a relatively young research field. The outcome of if a protein is mixed with a nanoparticle will depend on which protein it is and the material, size, shape and surface modification of the nanoparticle. In this review, we have summarized the knowledge gained so far on the interaction from one of the more studied proteins, hCA I and II, and model nanoparticles, silica and polystyrene.

The review also goes through many of the different methods that have been used in order to characterize the interaction/adsorption of proteins to nanoparticles. The review of the generated results from the studies of hCA I or hCA II interaction/adsorption to NPs illustrates the complexity of the research field. It will be hard to generate any general rules for predicting the outcome for mixing a protein with a nanoparticle. Despite the structure and enzymatic mechanism similarity of the two reviewed isoforms, hCA I and II, the adsorption process differs regarding driving forces, time scale, extend of conformational changes, etc. At the moment, every new protein–NP system has to be individually characterized. However, the knowledge gained for hCAs can be used to guide the characterization work that should preclude the design of a new bionanomaterial.

## Figures and Tables

**Table 1:** Type of nanoparticles and techniques used to study a particular aspect in the interaction between hCA I and hCA II with nanoparticles (NP).

Isoform	NP Type/Size (nm)	*Study*: Technique
**hCA I**	Silica/6–15 nm	*Adsorption process*: NMR [42,43,44], size exclusion chromatography [43], analytical ultracentrifugation [43] *Structural changes*: CD [43] *Enzymatic activity:* NPA activity assay [44]
	Silica/20–90 nm	*Adsorption process*: ANS fluorescence [45], Dynamic Light Scattering [4] *Structural changes:* ANS fluorescence [45]
	Carboxy-modified polystyrene/25–95 nm	*Adsorption process*: ANS fluorescence [45], activity assays [46], Dynamic Light Scattering [46], ITC [46] *Structural changes:* ANS fluorescence [45], CD [45] *Enzymatic activity:* NPA Activity assay [46]
	Gold/3 nm	*Adsorption process*: Bradford assay [47] *Enzymatic activity: NPA* activity assay [47]
	Fluorescence modified silica/3.5 nm	*Enzymatic activity:* CO_2_ hydration activity assay [18]
**hCA II** *	Silica/6–15 nm	*Adsorption process*: size exclusion chromatography [43,48] NMR [42,43], analytical ultracentrifugation [43], Ellipsometry [8], Scanning Differential Calorimetry [49] *Structural changes:* CD [43,50,51], MS [51], fluorescence [50] *Enzymatic activity:* NPA activity assay [48] *Binding site:* MS [51], fluorescence probes [52]
	Carboxy-modified polystyrene/25–115 nm	*Adsorption process*: ANS fluorescence [45], NPA activity assay [46], ITC [46,53], Dynamic Light Scattering [46] *Structural changes:* CD [45,53], ANS fluorescence [45] *Enzymatic activity:* NPA activity assay [46,53]
	Polystyrene/46 nm	*Adsorption process*: Activity assay [53], ITC [53] *Structural changes:* CD [53] *Enzymatic activity: NPA* activity assay [53]
	Amino modified polystyrene/49 nm	*Adsorption process*: NPA activity assay [53], ITC [53] *Structural changes:* CD [53] *Enzymatic activity:* NPA activity assay [53]
	Polymer modified Au/31–48 nm	*Adsorption process*: NPA activity assay [53], ITC [53] *Enzymatic activity:* NPA activity assay [53]
	Aminopropyl functionalized PEGylated mesoporous silica/75–80 nm	*Adsorption process*: Thermal Gravimetric Analysis [54], UV–vis spectroscopy [54], Differential Scanning Calorimetry [54] *Structural changes*: CD [54], fluorescence [54], *Enzymatic activity*: NPA activity assay [54]
	Fluorescence modified silica/3.5 nm	*Enzymatic activity:* CO_2_ hydration activity assay [18]
	Functionalized silica/100 nm	*Adsorption process*: Fluorescence [55], UV-vis spectroscopy [55], Differential Scanning Calorimetry [55]

* Pseudo-wild type, mutants and truncated variants of hCA II. Study in italics and Technique in normal font.

## References

[1] DiMario R.J., Machingura M.C., Waldrop G.L., Moroney J.V. (2018). The many types of carbonic anhydrases in photosynthetic organisms. Plant Sci..

[2] Del Prete S., Nocentini A., Supuran C.T., Capasso C. (2020). Bacterial ι-carbonic anhydrase: A new active class of carbonic anhydrase identified in the genome of the Gram-negative bacterium Burkholderia territorii. J. Enzym. Inhib. Med. Chem..

[3] Meldrum N.U., Roughton F.J.W. (1933). Carbonic anhydrase and the state of carbon dioxide in blood. Nature.

[4] Supuran C.T., Scozzafava A. (2007). Carbonic anhydrases as targets for medicinal chemistry. Biorg. Med. Chem..

[5] Supuran C.T. (2008). Carbonic anhydrases: Novel therapeutic applications for inhibitors and activators. Nat. Rev. Drug Discov..

[6] Kupriyanova E., Pronina N., Los D. (2017). Carbonic anhydrase—A universal enzyme of the carbon-based life. Photosynthetica.

[7] Schwirn K., Tietjen L., Beer I. (2014). Why are nanomaterials different and how can they be appropriately regulated under REACH?. Environ. Sci. Eur..

[8] Caruso F. (2001). Nanoengineering of particle surfaces. Adv. Mater. Processes.

[9] Georganopoulou D.G., Chang L., Nam J.M., Thaxton C.S., Mufson E.J., Klein W.L., Mirkin C.A. (2005). Nanoparticle-based detection in cerebral spinal fluid of a soluble pathogenic biomarker for Alzheimer’s disease. Proc. Natl. Acad. Sci. USA.

[10] Lynch I., Cedervall T., Lundqvist M., Cabaleiro-Lago C., Linse S., Dawson K.A. (2007). The nanoparticle - protein complex as a biological entity; a complex fluids and surface science challenge for the 21st century. Adv. Colloid Interface Sci..

[11] Matsumura S., Aoki I., Saga T., Shiba K. (2011). A Tumor-Environment-Responsive Nanocarrier That Evolves Its Surface Properties upon Sensing Matrix Metalloproteinase-2 and Initiates Agglomeration to Enhance T-2 Relaxivity for Magnetic Resonance Imaging. Mol. Pharm..

[12] Slocik J.M., Naik R.R. (2010). Probing peptide-nanomaterial interactions. Chem. Soc. Rev..

[13] Yong J.K.J., Stevens G.W., Caruso F., Kentish S.E. (2015). The use of carbonic anhydrase to accelerate carbon dioxide capture processes. J. Chem. Technol. Biotechnol..

[14] Yadav R.R., Krishnamurthi K., Mudliar S.N., Devi S.S., Naoghare P.K., Bafana A., Chakrabarti T. (2014). Carbonic anhydrase mediated carbon dioxide sequestration: Promises, challenges and future prospects. J. Basic Microbiol..

[15] Shekh A.Y., Krishnamurthi K., Mudliar S.N., Yadav R.R., Fulke A.B., Devi S.S., Chakrabarti T. (2012). Recent Advancements in Carbonic Anhydrase-Driven Processes for CO_2_ Sequestration: Minireview. Crit. Rev. Environ. Sci. Technol..

[16] Savile C.K., Lalonde J.J. (2011). Biotechnology for the acceleration of carbon dioxide capture and sequestration. Curr. Opin. Biotechnol..

[17] Stiti M., Cecchi A., Rami M., Abdaoui M., Barragan-Montero V., Scozzafava A., Guari Y., Winum J.-Y., Supuran C.T. (2008). Carbonic Anhydrase Inhibitor Coated Gold Nanoparticles Selectively Inhibit the Tumor-Associated Isoform IX over the Cytosolic Isozymes I and II. J. Am. Chem. Soc..

[18] Touisni N., Kanfar N., Ulrich S., Dumy P., Supuran C.T., Mehdi A., Winum J.-Y. (2015). Fluorescent Silica Nanoparticles with Multivalent Inhibitory Effects towards Carbonic Anhydrases. Chem. Eur. J..

[19] Innocenti A., Durdagi S., Doostdar N., Strom T.A., Barron A.R., Supuran C.T. (2010). Nanoscale enzyme inhibitors: Fullerenes inhibit carbonic anhydrase by occluding the active site entrance. Biorg. Med. Chem..

[20] Imtaiyaz Hassan M., Shajee B., Waheed A., Ahmad F., Sly W.S. (2013). Structure, function and applications of carbonic anhydrase isozymes. Biorg. Med. Chem..

[21] Lindskog S. (1997). Structure and mechanism of carbonic anhydrase. Pharmacol. Ther..

[22] Supuran C.T. (2016). Structure and function of carbonic anhydrases. Biochem. J..

[23] Supuran C.T. (2013). Carbonic anhydrases: From biomedical applications of the inhibitors and activators to biotechnological use for CO_2_ capture. J. Enzym. Inhib..

[24] Bhakta S.A., Evans E., Benavidez T.E., Garcia C.D. (2015). Protein adsorption onto nanomaterials for the development of biosensors and analytical devices: A review. Anal. Chim. Acta.

[25] Guler O.O., Capasso C., Supuran C.T. (2016). A magnificent enzyme superfamily: Carbonic anhydrases, their purification and characterization. J. Enzym. Inhib. Med. Chem..

[26] Khalifah R.G. (1971). The carbon dioxide hydration activity of carbonic anhydrase. I. Stop-flow kinetic studies on the native human isoenzymes B and C. J. Biol. Chem..

[27] Domsic J.F., Avvaru B.S., Kim C.U., Gruner S.M., Agbandje-McKenna M., Silverman D.N., McKenna R. (2008). Entrapment of Carbon Dioxide in the Active Site of Carbonic Anhydrase II. J. Biol. Chem..

[28] Freskgard P.O., Carlsson U., Martensson L.G., Jonsson B.H. (1991). Folding around the c-terminus of human carbonic anhydrase-II - kinetic characterization by use of a chemically reactive sh-group introduced by protein engineering. FEBS Lett..

[29] Carlsson U., Henderson L.E., Lindskog S. (1973). Denaturation and reactivation of human carbonic anhydrases in guanidine hydrochloride and urea. Biochim. Biophys. Acta.

[30] Carlsson U., Henderson L.E., Nyman P.O., Samuelsson T. (1974). Studies on influence of carboxyl-terminal amino-acid residues on activity and stability of human erythrocyte carbonic-anhydrase b. FEBS Lett..

[31] Carlsson U., Aasa R., Henderson L.E., Jonsson B.H., Lindskog S. (1975). Paramagnetic and fluorescent-probes attached to buried sulfhydryl groups in human carbonic-anhydrases - application to inhibitor binding, denaturation and refolding. Eur. J. Biochem..

[32] Kjellsson A., Sethson I., Jonsson B.H. (2003). Hydrogen exchange in a large 29 kD protein and characterization of molten globule aggregation by NMR. Biochemistry.

[33] Aronsson G., Martensson L.G., Carlsson U., Jonsson B.H. (1995). Folding and stability of the n-terminus of human carbonic-anhydrase-II. Biochemistry.

[34] Martensson L.G., Jonsson B.H., Freskgard P.O., Kihlgren A., Svensson M., Carlsson U. (1993). Characterization of folding intermediates of human carbonic-anhydrase.2. Probing substructure by chemical labeling of sh-groups introduced by site-directed mutagenesis. Biochemistry.

[35] Jagannadham M.V., Balasubramanian D. (1985). The molten globular intermediate form in the folding pathway of human carbonic anhydrase-B. FEBS Lett..

[36] Dolgikh D.A., Kolomiets A.P., Bolotina I.A., Ptitsyn O.B. (1984). ‘Molten-globule“ state accumulates in carbonic anhydrase folding. FEBS Lett..

[37] Martensson L.G., Jonasson P., Freskgard P.O., Svensson M., Carlsson U., Jonsson B.H. (1995). Contribution of individual tryptophan residues to the fluorescence-spectrum of native and denatured forms of human carbonic-anhydrase-II. Biochemistry.

[38] Andersson D., Freskgård P.O., Jonsson B.H., Carlsson U. (1997). Formation of local native-like tertiary structures in the slow refolding reaction of human carbonic anhydrase II as monitored by circular dichroism on tryptophan mutants. Biochemistry.

[39] Hagemans D., van Belzen I.A.E.M., Morán Luengo T., Rüdiger S.G.D. (2015). A script to highlight hydrophobicity and charge on protein surfaces. Front. Nurs. Serv. Q. Bull..

[40] Kannan K.K., Ramanadham M., Jones T.A. (1984). Structure, Refinement, and Function of Carbonic Anhydrase Isozymes: Refinement of Human Carbonic Anhydrase I. Ann. N. Y. Acad. Sci..

[41] Fisher S.Z., Maupin C.M., Budayova-Spano M., Govindasamy L., Tu C., Agbandje-McKenna M., Silverman D.N., Voth G.A., McKenna R. (2007). Atomic Crystal and Molecular Dynamics Simulation Structures of Human Carbonic Anhydrase II:  Insights into the Proton Transfer Mechanism. Biochemistry.

[42] Lundqvist M., Sethson I., Jonsson B.H. (2005). High-resolution 2D H-1-N-15 NMR characterization of persistent structural alterations of proteins induced by interactions with silica nanoparticles. Langmuir.

[43] Lundqvist M., Sethson I., Jonsson B.H. (2004). Protein Adsorption onto Silica Nanoparticles: Conformational Changes Depend on the Particles’ Curvature and Protein Stability. Langmuir.

[44] Lundqvist M., Sethson I., Jonsson B.H. (2005). Transient interaction with nanoparticles "freezes" a protein in an ensemble of metastable near-native conformations. Biochemistry.

[45] Nasir I., Lundqvist M., Cabaleiro-Lago C. (2015). Size and surface chemistry of nanoparticles lead to a variant behavior in the unfolding dynamics of human carbonic anhydrase. Nanoscale.

[46] Assarsson A., Nasir I., Lundqvist M., Cabaleiro-Lago C. (2016). Kinetic and thermodynamic study of the interactions between human carbonic anhydrase variants and polystyrene nanoparticles of different size. Rsc Advances.

[47] Vinoba M., Lim K.S., Lee S.H., Jeong S.K., Alagar M. (2011). Immobilization of Human Carbonic Anhydrase on Gold Nanoparticles Assembled onto Amine/Thiol-Functionalized Mesoporous SBA-15 for Biomimetic Sequestration of CO_2_. Langmuir.

[48] Karlsson M., Mårtensson L.G., Jonsson B.H., Carlsson U. (2000). Adsorption of human carbonic anhydrase II variants to silica nanoparticles occur stepwise: Binding is followed by successive conformational changes to a molten-globule-like state. Langmuir.

[49] Billsten P., Carlsson U., Jonsson B.H., Olofsson G., Höök F., Elwing H. (1999). Conformation of human carbonic anhydrase II variants adsorbed to silica nanoparticles. Langmuir.

[50] Billsten P., Freskgård P.O., Carlsson U., Jonsson B.H., Elwing H. (1997). Adsorption to silica nanoparticles of human carbonic anhydrase II and truncated forms induce a molten-globule-like structure. FEBS Lett..

[51] Lundqvist M., Andresen C., Christensson S., Johansson S., Karlsson M., Broo K., Jonsson B.H. (2005). Proteolytic Cleavage Reaveals Interacton Patterns between Silica Nanoparticles and Two Variants of Human Carbonic Anhydrase. Langmuir.

[52] Karlsson M., Carlsson U. (2005). Protein adsorption orientation in the light of fluorescent probes: Mapping of the interaction between site-directly labeled human carbonic anhydrase II and silica nanoparticles. Biophys. J..

[53] Assarsson A., Pastoriza-Santos I., Cabaleiro-Lago C. (2014). Inactivation and Adsorption of Human Carbonic Anhydrase II by Nanoparticles. Langmuir.

[54] Khatibi A., Ma’mani L., Khodarahmi R., Shafiee A., Maghami P., Ahmad F., Sheibani N., Moosavi-Movahedi A.A. (2015). Enhancement of thermal reversibility and stability of human carbonic anhydrase II by mesoporous nanoparticles. Int. J. Biol. Macromol..

[55] Fallah-Bagheri A., Saboury A.A., Ma’mani L., Taghizadeh M., Khodarahmi R., Ranjbar S., Bohlooli M., Shafiee A., Foroumadi A., Sheibani N. (2012). Effects of silica nanoparticle supported ionic liquid as additive on thermal reversibility of human carbonic anhydrase II. Int. J. Biol. Macromol..

[56] Ranjbar B., Gill P. (2009). Circular Dichroism Techniques: Biomolecular and Nanostructural Analyses- A Review. Chem. Biol. Drug Des..

[57] Freskgard P.O., Maartensson L.G., Jonasson P., Jonsson B.H., Carlsson U. (1994). Assignment of the contribution of the tryptophan residues to the circular-dichroism spectrum of human carbonic-anhydrase II. Biochemistry.

[58] Sethson I., Edlund U., Holak T.A., Ross A., Jonsson B.H. (1996). Sequential assignment of H-1, C-13 and N-15 resonances of human carbonic anhydrase I by triple-resonance NMR techniques and extensive amino acid-specific N-15-labeling. J. Biomol. NMR.

[59] Venters R.A., Farmer B.T., Fierke C.A., Spicer L.D. (1996). Characterizing the use of perdeuteration in NMR studies of large proteins C-13, N-15 and H-1 assignments of human carbonic anhydrase II. J. Mol. Biol..

[60] Riek R., Pervushin K., Wüthrich K. (2000). TROSY and CRINEPT: NMR with large molecular and supramolecular structures in solution. Trends Biochem. Sci.

[61] Hawe A., Sutter M., Jiskoot W. (2008). Extrinsic fluorescent dyes as tools for protein characterization. Pharm. Res..

[62] Nasir I., Fatih W., Svensson A., Radu D., Linse S., Lago C.C., Lundqvist M. (2015). High Throughput Screening Method to Explore Protein Interactions with Nanoparticles. PLoS ONE.

[63] Manokaran S., Zhang X., Chen W., Srivastava D.K. (2010). Differential modulation of the active site environment of human carbonic anhydrase XII by cationic quantum dots and polylysine. Biochim. Biophys. Acta.

[64] Pocker Y., Stone J.T. (1967). Catalytic versatility of erythrocyte carbonic anhydrase.3. Kinetic studies of enzyme-catalyzed hydrolysis of p-nitrophenyl acetate. Biochemistry.

[65] Verpoorte J.A., Mehta S., Edsall J.T. (1967). Esterase activities of human carbonic anhydrases B and C. J. Biol. Chem..

[66] Prozeller D., Morsbach S., Landfester K. (2019). Isothermal titration calorimetry as a complementary method for investigating nanoparticle–protein interactions. Nanoscale.

[67] Krishnamurthy V.M., Kaufman G.K., Urbach A.R., Gitlin I., Gudiksen K.L., Weibel D.B., Whitesides G.M. (2008). Carbonic anhydrase as a model for biophysical and physical-organic studies of proteins and protein-ligand binding. Chem. Rev..

[68] Zhang X.N., Zhang J.T., Zhang F., Yu S.N. (2017). Probing the binding affinity of plasma proteins adsorbed on Au nanoparticles. Nanoscale.

[69] Zhang H., Wu R.a. (2015). Proteomic profiling of protein corona formed on the surface of nanomaterial. Sci. China Chem..

[70] Buijs J., Ramstrom M., Danfelter M., Larsericsdotter H., Hakansson P., Oscarsson S. (2003). Localized changes in the structural stability of myoglobin upon adsorption onto silica particles, as studied with hydrogen/deuterium exchange mass spectrometry. J. Colloid Interface Sci..

[71] Larsericsdotter H., Oscarsson S., Buijs J. (2005). Structure, stability, and orientation of BSA adsorbed to silica. J. Colloid Interface Sci..

[72] Norde W. (1992). The behavior of proteins at interfaces, with special attention to the role of the structure stability of the protein molecule. Clin. Mater..

[73] Haynes C.A., Norde W. (1995). Structure and stabilities of adsorbed proteins. J. Colloid Interface Sci..

[74] Tripp B.C., Magda J.J., Andrade J.D. (1995). Adsorption of globular-proteins at the air/water interface as measured via dynamic surface-tension - concentration-dependence, mass-transfer considerations, and adsorption-kinetics. J. Colloid Interface Sci..

[75] Ptitsyn O.B., Pain R.H., Semisotnov G.V., Zerovnik E., Razgulyaev O.I. (1990). Evidence for a molten globule state as a general intermediate in protein folding. FEBS Lett..

[76] Semisotnov G.V., Rodionova N.A., Razgulyaev O.I., Uversky V.N., Gripas A.F., Gilmanshin R.I. (1991). Study of the molten globule intermediate state in protein folding by a hydrophobic fluorescent-probe. Biopolymers.

[77] Patil S., Reshetnikov S., Haldar M.K., Seal S., Mallik S. (2007). Surface-derivatized nanoceria with human carbonic anhydrase II inhibitors and fluorophores: A potential drug delivery device. J. Phys. Chem. C Nanomater. Interfaces.

[78] Saada M.C., Montero J.L., Vullo D., Scozzafava A., Winum J.Y., Supuran C.T. (2011). Carbonic Anhydrase Activators: Gold Nanoparticles Coated with Derivatized Histamine, Histidine, and Carnosine Show Enhanced Activatory Effects on Several Mammalian Isoforms. J. Med. Chem..

